# Retraction: Bruder, L. et al. Transcatheter Decellularized Tissue-Engineered Heart Valve (dTEHV) Grown on Polyglycolic Acid (PGA) Scaffold Coated with P4HB Shows Improved Functionality over 52 Weeks due to Polyether-Ether-Ketone (PEEK) Insert. *J. Funct. Biomater*. 2018, *9*(4), 64

**DOI:** 10.3390/jfb10010008

**Published:** 2019-01-20

**Authors:** Leon Bruder, Kerstin Brakmann, Valentin Stegner, Matthias Sigler, Felix Berger, Boris Schmitt

**Affiliations:** 1 Deutsches Herzzentrum Berlin, Department of Congenital Heart Disease, 13353 Berlin, Germany; kbrakmann@gmx.de (K.B.); valentin.stegner@charite.de (V.S.); berger@dhzb.de (F.B.); schmitt@dhzb.de (B.S.); 2 Universitätsmedizin Göttingen, Herzzentrum Göttingen, Department of Pediatric Cardiology, 37075 Göttingen, Germany; msigler@gwdg.de

Due to human error, the authors included some of the experimental data in this article [1] which have already been published before by Emmert et al. [2], as well as Schmitt et al [3]. In addition, consent to publish from consortium partners was not properly received prior to publication. We have therefore requested that the paper is retracted.

MDPI is a member of the Committee on Publication Ethics and takes the responsibility to enforce strict ethical policies and standards very seriously. To ensure the addition of only high quality scientific works to the field of scholarly publication, [1] is retracted and shall be marked accordingly. The article is retracted with the agreement of all authors. We apologize to the readership of Journal of Functional Biomaterials for any inconvenience caused.
